# Folding heterogeneity in the essential human telomerase RNA three-way junction

**DOI:** 10.1261/rna.077255.120

**Published:** 2020-12

**Authors:** Christina Palka, Nicholas M. Forino, Jendrik Hentschel, Rhiju Das, Michael D. Stone

**Affiliations:** 1 Department of Chemistry and Biochemistry, University of California, Santa Cruz, California 95064, USA; 2 Department of Molecular, Cell, and Developmental Biology, University of California, Santa Cruz, California 95064, USA; 3 Biophysics Program, Stanford University, Stanford, California 94305, USA; 4 Department of Biochemistry, Stanford University, Stanford, California 94305, USA; 5 Department of Physics, Stanford University, Stanford, California 94305, USA; 6 Center for Molecular Biology of RNA, University of California, Santa Cruz, California 95064, USA

**Keywords:** RNA folding, telomerase, single-molecule FRET, structure modeling

## Abstract

Telomeres safeguard the genome by suppressing illicit DNA damage responses at chromosome termini. To compensate for incomplete DNA replication at telomeres, most continually dividing cells, including many cancers, express the telomerase ribonucleoprotein (RNP) complex. Telomerase maintains telomere length by catalyzing de novo synthesis of short DNA repeats using an internal telomerase RNA (TR) template. TRs from diverse species harbor structurally conserved domains that contribute to RNP biogenesis and function. In vertebrate TRs, the conserved regions 4 and 5 (CR4/5) fold into a three-way junction (TWJ) that binds directly to the telomerase catalytic protein subunit and is required for telomerase function. We have analyzed the structural properties of the human TR (hTR) CR4/5 domain using a combination of in vitro chemical mapping, secondary structural modeling, and single-molecule structural analysis. Our data suggest the essential P6.1 stem–loop within CR4/5 is not stably folded in the absence of the telomerase reverse transcriptase in vitro. Rather, the hTR CR4/5 domain adopts a heterogeneous ensemble of conformations. Finally, single-molecule FRET measurements of CR4/5 and a mutant designed to stabilize the P6.1 stem demonstrate that TERT binding selects for a structural conformation of CR4/5 that is not the dominant state of the TERT-free in vitro RNA ensemble.

## INTRODUCTION

The ends of linear chromosomes in eukaryotic cells terminate with repetitive DNA sequences that bind to specialized proteins to form telomeres (Blackburn and Gall 1978; Erdel et al. 2017). Telomeres protect coding DNA from degradation and distinguish chromosomal termini from double-stranded breaks to evade unwanted recognition by DNA damage response machineries (Muller 1938; McClintock 1939; de Lange 2018). With each round of cell division, the inability of the conventional replication machinery to completely copy the lagging strand template results in gradual telomere attrition. Ultimately the presence of a critically short telomere drives cells into permanent cell growth arrest or apoptosis (Hayflick 1965; Harley et al. 1990). However, cells that must retain high proliferative capacity maintain telomere length through the action of the telomerase reverse transcriptase (Greider and Blackburn 1985, 1989; Kolquist et al. 1998; Wright et al. 2001; Roth et al. 2003). Given the importance of maintaining telomere length in dividing cells, germ-line mutations in telomerase genes result in severe developmental defects (Yamaguchi et al. 2003; Vulliamy and Dokal 2008; Savage 2014). In addition, telomerase contributes to the unchecked cell growth that is a hallmark of human cancers (Kim et al. 1994; Blasco 2005). Therefore, efforts to better understand telomerase structure, function, and regulation have direct biomedical significance.

**FIGURE 1.**
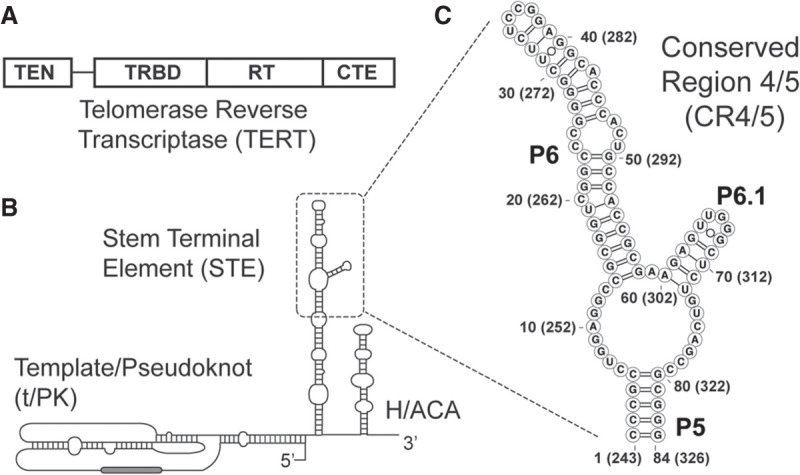
Conserved protein and RNA domains of the telomerase catalytic core. (*A*) The conserved domain architecture of the telomerase reverse transcriptase (TERT) catalytic protein subunit, including the telomerase essential amino-terminal (TEN) domain, the RNA-binding domain (TRBD), the reverse transcriptase (RT) domain, and the carboxy-terminal extension (CTE). (*B*) The conserved domain organization of the human telomerase RNA (TR), including the template/pseudoknot (t/PK) domain, the stem terminal element (STE), and the H/ACA box motif. (*C*) Conserved regions 4 and 5 (CR4/5) domain of the human TR (hTR) comprised of stems P5, P6, and P6.1. Nucleotide numbering system used throughout the study is indicated together with the corresponding nucleotide numbering within full-length hTR in parentheses.

Telomerase is a multisubunit ribonucleoprotein (RNP) complex that includes the catalytic telomerase reverse transcriptase (TERT) protein, telomerase RNA (TR), and several additional species-specific holoenzyme proteins that are necessary for proper RNP biogenesis (Egan and Collins 2012a; Chan et al. 2017). The TERT domain architecture is well-conserved across species and consists of the telomerase essential amino-terminal (TEN) domain, the telomerase RNA-binding domain (TRBD), the reverse transcriptase (RT) domain, and the carboxy-terminal extension (CTE) (Fig. 1A). In contrast, comparison of TRs across species ranging from yeasts to human reveals an exceedingly high degree of variation in both RNA length and sequence (Romero and Blackburn 1991; Chen et al. 2000; Chen and Greider 2004). Interestingly, in spite of this apparent evolutionary divergence, several conserved TR structural elements exist that are essential for enzyme assembly and function. These include the highly conserved template/pseudoknot (t/PK) domain and a stem-terminal element (STE) (Fig. 1B). In vertebrate TRs, the STE is thought to fold into an RNA three-way junction (TWJ) often referred to as the conserved regions 4/5 (CR4/5) domain (Fig. 1C). With regard to TR primary sequence, the CR4/5 domain is spatially separated from the RNA template that must necessarily reside in the TERT enzyme active site; yet, naturally occurring mutations in human telomerase RNA (hTR) CR4/5 can result in human diseases characterized by loss of telomerase function (Yamaguchi et al. 2003; Vulliamy and Dokal 2008; Alder et al. 2018).

In hTR, the CR4/5 domain includes three RNA helices (P5, P6, and P6.1) joined together by an expanded RNA junction sequence (Fig. 1C). Detailed biochemical studies performed on vertebrate TR CR4/5 variants have shown that a stably formed P6.1 helix within the TWJ is essential for telomerase assembly and function (Mitchell and Collins 2000; Chen et al. 2002; Kim et al. 2014). Chemical and enzymatic RNA structure probing experiments of full-length hTR have reported a complex pattern of both reactivity and protection in the P6.1 stem and the adjacent junction region leading to mixed conclusions regarding the overall architecture of the TWJ region (Antal et al. 2002; Zemora et al. 2016). However, NMR studies of isolated P6.1 constructs have demonstrated that this RNA sequence is capable of adopting a stable stem–loop motif and is even further stabilized by pseudouridine modifications that may occur in some hTR molecules in vivo (Leeper and Varani 2005; Kim et al. 2010; Zemora et al. 2016). More recently, the human telomerase holoenzyme protein TCAB1 was implicated in mediating proper folding of the CR4/5 TWJ domain (Chen et al. 2018). Protein–RNA cross-linking studies and an atomic-resolution structure of the medaka fish TR TWJ bound by its cognate TERT–TRBD revealed the molecular details of the TERT–RNA interaction (Bley et al. 2011; Kim et al. 2014). Interestingly, the helical arrangement observed in the medaka protein–RNA complex was substantially altered when compared to the solution structure of the same RNA domain in the absence of protein (Huang et al. 2014). Over the last several years, cryo-EM structures of the *Tetrahymena* and human telomerase RNPs were reported (Jiang et al. 2018; Nguyen et al. 2018), providing additional details on the arrangement of protein and RNA domains within the fully assembled telomerase RNP complex. Both structures suggest that an apical stem–loop within the STE (P6.1 in hTR) lies at the interface of the TERT–CTE and TERT–TRBD domains, providing clues as to the essential requirement of the P6.1 stem–loop in coupling the two TERT domains during telomerase assembly and/or function. Despite significant advances in structural studies on hTR, open questions remain regarding the predominant fold and stability of CR4/5 in its RNP unbound state and how the folding of this junction changes upon RNP assembly.

Here, we set out to characterize the in vitro RNA folding properties of the hTR CR4/5 domain using a combination of chemical mapping and structural modeling, paired together with single-molecule Förster resonance energy transfer (smFRET) experiments. Chemical probing experiments using a variety of RNA modification reagents revealed a substantial degree of reactivity within the region of hTR CR4/5 expected to form the essential P6.1 stem–loop structure. Use of chemical reactivity data to guide computational modeling of CR4/5 structure reveals the hTR P6.1 stem is predicted to fold with much less confidence than the medaka P6.1 stem. To further characterize hTR CR4/5 structure, we systematically perturbed each nucleotide within the hTR CR4/5 domain, and queried the effects of each mutation on the chemical reactivity profile (Kladwang et al. 2011; Tian et al. 2014). The results of these multidimensional chemical mapping (MCM) experiments reinforce the conclusion that the P6.1 stem–loop is not well ordered in vitro. Our use of smFRET to probe the tertiary conformational properties of the hTR CR4/5 domain revealed its heterogeneous RNA folding behavior, characterized by at least three distinct FRET states. The FRET profile of a CR4/5 mutant engineered to stabilize the canonical secondary structure of the P6.1 stem was comparatively enriched with a low FRET state, and the WT CR4/5 bound to TERT yielded a homogenous FRET profile consisting of a similar low FRET state. Collectively, our results suggest the majority of molecules in the in vitro CR4/5 structural ensemble do not possess a stably folded P6.1. Upon binding TERT, CR4/5 structural heterogeneity is suppressed and the domain adopts a more uniform conformation, likely the canonical TWJ including the essential P6.1 stem.

## RESULTS

### Chemical probing of the telomerase RNA three-way junction

The TWJ motif is well conserved across many telomerase RNA systems, ranging from yeasts to vertebrates. Many of the RNA structural models that are used to generate hypotheses relating to telomerase function are derived from sequence covariation analysis (Chen and Greider 2004) and/or the use of biochemical mutagenesis (Mitchell and Collins 2000; Chen et al. 2002). One challenge of methods such as sequence covariation analysis is that the resultant models may not accurately capture the structural properties of all RNA folding intermediates before it interacts with physiological binding partners. Indeed, studies of telomerase biogenesis indicate that hTR accumulates in subnuclear compartments prior to assembly with the TERT protein subunit (Etheridge et al. 2002; Zhu et al. 2004), raising the distinct possibility that hTR may exist in various structural states prior to telomerase assembly. To better understand the structural properties of TRs prior to and during RNP biogenesis, we set out to analyze the secondary structural properties of telomerase TWJs from two vertebrate systems: medaka fish (*Oryzias latipes*) and human. The medaka TR TWJ serves as an important benchmark in our TR structural analyses because its atomic structure is well characterized in the absence and presence of the TERT–TRBD (Huang et al. 2014; Kim et al. 2014).

**FIGURE 2.**
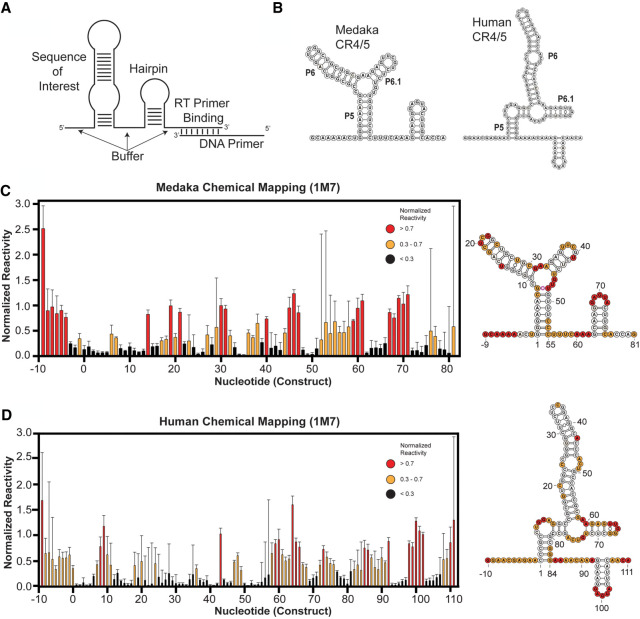
Chemical mapping of medaka and human CR4/5 domains. (*A*) Cartoon schematic of general RNA construct design, including the RNA sequence of interest flanked by unstructured RNA buffer sequences, a normalization RNA hairpin, and a reverse transcriptase priming site. (*B*) Lowest energy predicted secondary structure of medaka (*left*) and human (*right*) CR4/5 domain using RNAstructure. (*C*) (*left*) Chemical mapping of the medaka CR4/5 domain by SHAPE (1M7 probing) at 1 mM MgCl_2_. (*D*) Chemical mapping of the human CR4/5 domain by SHAPE (1M7 probing) at 1 mM MgCl_2_. For both (*C*,*D*), color coding in the bar plot and structure schematic is as described in *C*. Plotted normalized reactivity values are color-coded (red >0.7, yellow 0.3–0.7, and black <0.3). Each bar plotted represents experiments conducted in triplicate or greater with the respective standard deviation as error bars (*right*). Color-coded schematic of the reactivity data is shown on the RNAstructure predicted secondary structure.

For each TR system, we used an isolated CR4/5 RNA fragment to facilitate in vitro structure probing. Notably, the isolated hTR CR4/5 domain used in our studies is sufficient to support telomerase function when reconstituted with the hTR t/PK domain and TERT protein (Supplemental Fig. S1; Tesmer et al. 1999). Several sequence elements were added to the TR segment to assist in quantitative data analysis of chemical probing experiments (Fig. 2A). First, a primer binding site was appended to the RNA 3′-end for use in the reverse transcriptase reactions required to readout sites of RNA modification. Second, a short RNA hairpin structure flanked by unstructured “buffer” regions was added to serve as an internal normalization control when calculating chemical reactivities (see Materials and Methods for details) (Kladwang et al. 2014). De novo structure predictions using only the RNA primary sequences as calculated on the RNAstructure web server (Reuter and Mathews 2010) yielded the lowest free energy conformations with the expected stems that collectively form the TWJ fold (Fig. 2B). In the case of the hTR CR4/5 domain, RNAstructure predicted an additional cross-junction clamping helix not typically included in canonical representations of this region of hTR. Furthermore, multiple structures with nearly isoenergetic stability were also predicted, including conformations lacking the essential P6.1 stem–loop (Supplemental Fig. S2), highlighting the need for experimental data to validate specific RNA models.

To experimentally evaluate each of these CR4/5 structure predictions, we performed selective hydroxyl acylation analyzed by primer extension (SHAPE) experiments using 1-methyl-7-nitroisatoic anhydride (1M7), a fast-acting chemical modifier (Mortimer et al. 2012; Turner et al. 2013). In addition, experiments were also performed using the base-specific reagents dimethyl sulfate (DMS) or 1-cyclohexyl-(2-morpholinoethyl) carbodiimide metho-*p*-toluene sulfonate (CMCT), which primarily react with adenine/cytosine or guanine/uracil bases, respectively (Supplemental Fig. S3). Reactivity profiles obtained by all three chemical probing methods (DMS, CMCT, and 1M7) for the medaka CR4/5 yielded data that support the canonical base pairing arrangement expected for this TWJ fold, and are highly consistent with the reported solution structure of this same RNA fragment (Fig. 2C; Supplemental Figs. S3 and S4; Kim et al. 2014). In contrast, for the human CR4/5 domain, strong 1M7 reactivity was observed in the region expected to fold into the P6.1 stem (Fig. 2D). To test whether this discrepancy in SHAPE profiles of the human and medaka CR4/5 domains was due to unique structural interactions with magnesium, the SHAPE experiments were repeated across a titration of MgCl_2_. Interestingly, the reactivity patterns did not show any detectable MgCl_2_ dependence for either the medaka or human construct (Supplemental Fig. S5). The reactivity observed in the hTR P6.1 stem is unexpected given previous structural studies of isolated P6.1 constructs (Leeper and Varani 2005) and the established importance of the P6.1 stem–loop structure in promoting telomerase RNP assembly and function (Mitchell and Collins 2000; Chen et al. 2002; Kim et al. 2014) but is consistent with previous studies that use chemical mapping to examine the CR4/5 in full-length hTR in vivo and in vitro (Antal et al. 2002; Zemora et al. 2016). Taken together, these data suggest that using primary sequence information alone, the RNAstructure folding algorithm effectively predicts a base pairing configuration suggested by the SHAPE data of the medaka TR TWJ. However, significant disparity between the sequence alone prediction and the SHAPE data are observed in the expanded junction/6.1 stem of the hTR CR4/5 domain. Thus, human CR4/5 displays a complex folding behavior that confounds RNAstructure predictions in the absence of chemical probing data.

### SHAPE-guided modeling of human CR4/5 does not support formation of the P6.1 stem

RNAstructure calculates the lowest free energy structures using thermodynamic parameters that are dynamically sampled against databases of structures with well-characterized stabilities (Reuter and Mathews 2010). Experimentally derived chemical probing data significantly improves the predictive power of the RNAstructure folding algorithm (Mathews et al. 2004). SHAPE reactivities are used to calculate a pseudoenergy change term (ΔG_SHAPE_) at each nucleotide *i* using the formula ΔG_SHAPE_(*i*) = *m* ln(SHAPE reactivity(*i*) + 1) + *b*, which is then utilized as a nearest neighbor free energy term for structure prediction (Deigan et al. 2009). The slope and intercept parameters *m* and *b*, respectively, were empirically parameterized against the 23S rRNA and produce accurate (>89% correct base pairs) predictions even when varied within a large “sweet spot” of absolute values (Deigan et al. 2009). Importantly, the slope parameter *m* can be increased to disfavor the prediction of helices containing reactive nucleotides.

Using this approach, we performed SHAPE experiments of the previously mentioned medaka and human CR4/5 constructs, then generated SHAPE-guided structure models while increasing the slope parameter within its accurate range (1.8–5 kcal/mol). In our analysis, we used the Biers component of the HiTRACE software package to implement RNAstructure with a nonparametric bootstrapping function to estimate confidence values for each RNA helix in the predicted structures (Kladwang et al. 2011; Tian et al. 2014). The bootstrapping function iteratively subsamples the reactivity data with replacement, then runs the RNAstructure algorithm. The collection of bootstrapping-derived structures is then used to calculate the frequency of each RNA helix present across all computationally derived replicates. In this way, the resulting bootstrap value for any given helix provides a metric to evaluate its predictive confidence. It is important to note that bootstrap values are a statistical tool to analyze computational prediction methods, and should not be interpreted as an indicator of the equilibrium conformation(s) present for a particular RNA of interest.

**FIGURE 3.**
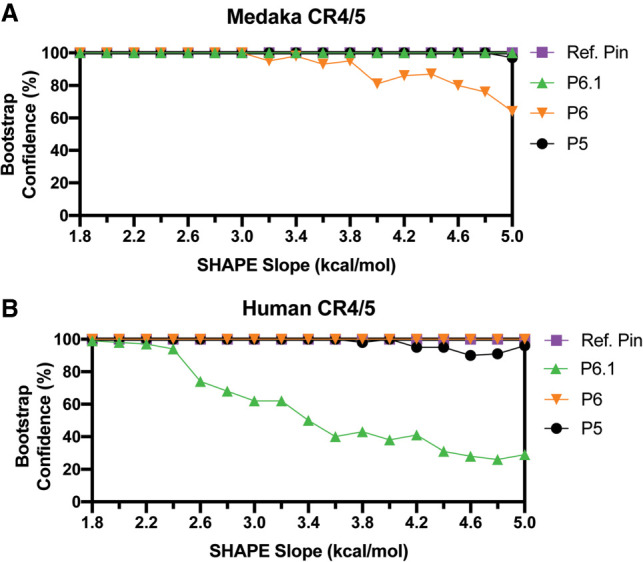
Data-guided RNA secondary structure prediction of medaka and human CR4/5 domain. SHAPE (1M7) reactivity data were used as weights to guide RNA structure prediction for medaka (*A*) and human (*B*) CR4/5 domains. Using the Biers package of HiTRACE, RNAstructure models of each RNA domain were calculated with 100 bootstrap replicates, while varying the SHAPE slope parameter in intervals of 0.2 kcal/mol. The abundance of each helical RNA element (Confidence) derives from the bootstrap replicates and is plotted for each respective value of SHAPE slope. Ref. Pin refers to Reference hairpin.

As expected, the addition of the ΔG_SHAPE_ constraints to predictions of the medaka TR CR4/5 yields the canonical TWJ fold with each of the expected helices being called with high confidence as the SHAPE slope parameter was increased (Fig. 3A). Bootstrap-calculated confidence in the P6 stem slightly decreases at higher SHAPE slope (>4 kcal/mol) because of the presence of moderate SHAPE reactivity at nucleotides known to be base paired in the crystal structure (G14, C16, A17). Overall, this result indicated that addition of experimentally derived data does not cause the RNAstructure algorithm to significantly deviate in its prediction of the lowest energy conformation for the medaka TR CR4/5. In the case of the hTR CR4/5, the inclusion of ΔG_SHAPE_ constraints in structure calculation recaptures a lowest energy conformation in which the P5, P6, and normalization hairpin are called with high confidence. In contrast, the confidence value of the P6.1 stem significantly decreases as SHAPE slope increases, consistent with the high levels of SHAPE reactivity in this region disfavoring the prediction of a stem–loop motif (Fig. 3B). These data-driven structure predictions indicate the hTR CR4/5 domain likely does not adopt its expected TWJ motif in the absence of telomerase-associated proteins in vitro.

### Multidimensional chemical mapping supports hTR CR4/5 structural heterogeneity

**FIGURE 4.**
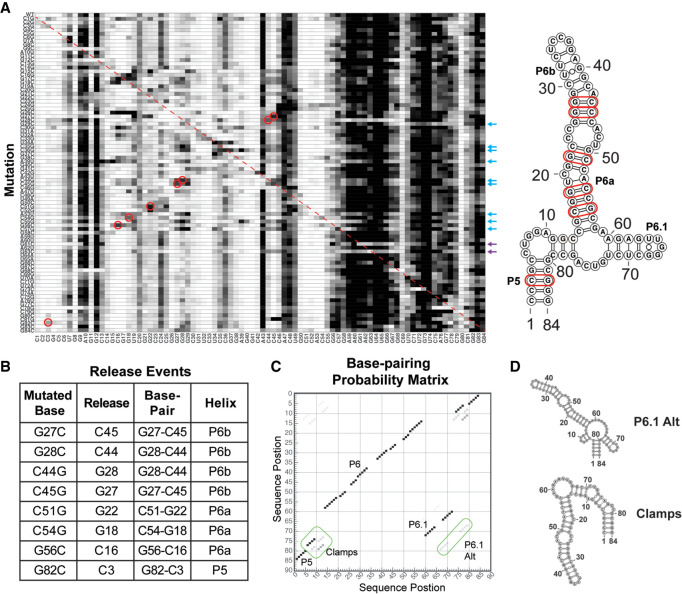
Mutate-and-map profiling of the human CR4/5 domain indicates the presence of structural heterogeneity within the RNA junction region. (*A*) Systematic mutations were introduced at each base within the hTR CR4/5 domain as indicated (A → U, U → A, G → C, and C → U). The structure of each mutant was interrogated by SHAPE (1M7), and the resultant reactivity profiles were stacked to create a reactivity tapestry that permits visual comparison of the chemical reactivity at each nucleotide across all mutants. The red dashed line corresponds to the position of expected signal of enhanced reactivity at the site of the base substitution. Specific sites of enhanced reactivity (“release events”) are circled in red. Positions of validated base pairing interactions are highlighted in red in the secondary structure model shown to the right. Mutation positions with the P6 stem (blue arrows) and P6.1 stem (purple arrows) that induce large-scale changes in the reactivity patterns are indicated. (*B*) Summary of specific mutations and sites of correlated enhancements of chemical reactivity together with the positions of the CR4/5 base pairs that these data support. (*C*) Bootstrap support values are plotted in a base pair probability matrix represented in gray scale. High confidence stems give rise to dark and symmetric signals. Each of the RNA structure elements are annotated including noncanonical cross-junction clamps and an alternate P6.1 stem. (*D*) Representative alternative hTR CR4/5 junction structure predictions from mutate-and-map experiments.

To further probe the structure of the hTR CR4/5 domain, we performed multidimensional chemical mapping (MCM) (Kladwang et al. 2011). This systematic mutagenesis approach permits rapid chemical probing analysis of a panel of RNA mutant constructs designed to explicitly test for the presence of Watson–Crick base pairing in a proposed RNA secondary structural model (Kladwang et al. 2011; Tian et al. 2014). If a mutation is made to a base that is engaged in a base pair, then one expects the release of the interacting partner that consequently becomes accessible to the SHAPE probe. To probe for such specific release events, we generated a set of 84 mutants across the entire hTR CR4/5 construct. The chemical reactivity profiles of all RNA variants were stacked vertically to generate a reactivity tapestry (Fig. 4A). Signals on the diagonal of the reactivity tapestry represent release events at the engineered site of mutation (Fig. 4A, red dotted line). Signals that deviate from the wild-type reactivity profile indicate changes in reactivity that result from each individual mutation. Many of the single-mutant reactivity profiles revealed complex structural rearrangements beyond the simple base pair release event principle. However, visual inspection of the data reveals multiple features in the reactivity tapestry that support specific base pairs present within the hTR CR4/5 (Fig. 4A, red circles, and Fig. 4B). For example, the G27C and G28C mutations each resulted in increased reactivity at positions C45 and C44, respectively, providing support for these base pairs being present within the P6b stem (Fig. 4B). Similarly, the C44G and C45G mutations resulted in release events in G28 and G27, respectively, providing independent support for these same base pairs in the P6b stem. Increased reactivity was also observed for certain mutations within the P6a stem; for example, the C51G, C54G, and G56C mutations each caused increased signal at positions G22, G18, and C16, respectively. Lastly, the G82C mutation located within the P5 stem resulted in increased reactivity at position C3, providing support for this specific base pairing interaction. Notably, the high baseline reactivity observed in the hTR CR4/5 junction and P6.1 stem–loop region precludes unambiguous visual analysis of the MCM data. However, we found that mutations introduced at the base of the P6a stem (A53U, C55G, and C57G) and several mutations in P6b had the unexpected effect of causing substantial structural rearrangements in the CR4/5 domain, evidenced by reduced reactivity in the junction region and increased reactivity within the P6 stem (Fig. 4A, blue arrows). Other notable global folding changes were observed for single G → C substitutions located within the P6.1 stem, such as G61C and G63C, which both induce the CR4/5 domain to fold into an extended two-helix junction (Fig. 4A, purple arrows, and Supplemental Fig. S6).

To achieve a quantitative analysis across the entire reactivity tapestry we generated a *Z*-score plot, where individual *Z*-scores report on the statistical significance of deviations in the reactivity level for a given nucleotide compared across all RNA constructs (Supplemental Fig. S7). *Z*-score values are then used as a pseudoenergy term to guide structure prediction by RNAstructure within the Biers component of the HiTRACE software package (Tian et al. 2014). As with the SHAPE reactivity-guided RNAstructure calculations, the *Z*-score data can be used to perform bootstrapping analysis as a measure of confidence in each predicted helical segment and to generate a base pair probability matrix (Fig. 4C). The results of the *Z*-score analysis are consistent with the presence of structures other than the canonical P5, P6, and P6.1 stems in the CR4/5 structure ensemble. For example, in multiple Z-score-driven structures, an alternative P6.1 stem (P6.1 alt) was predicted in addition to several mutually exclusive cross-junction clamping helices (Fig. 4D). Taken together, the results of the MCM experiments provide additional experimental evidence for base pairing interactions in the P6a, P6b, and P5 stems, and support the notion that the junction region and P6.1 stem–loop may adopt noncanonical base pairing configurations.

### Single-molecule analysis reveals CR4/5 folding heterogeneity and remodeling upon telomerase RNP assembly

**FIGURE 5.**
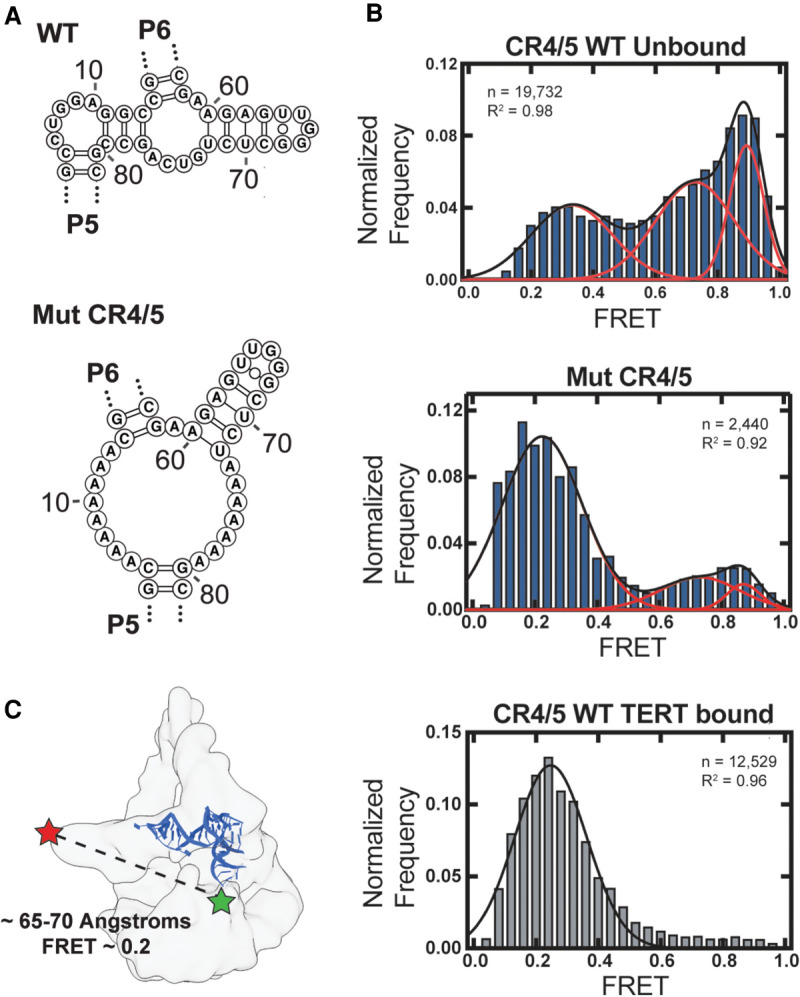
Single-molecule FRET analysis of hTR CR4/5 domain. (*A*) RNAstructure predicted junction structures for WT hTR CR4/5 (*top* panel) and Mut CR4/5 (*middle* panel). (*B*) Histograms of smFRET data collected in the presence of 1 mM MgCl_2_ using a confocal microscope of freely diffusing CR4/5 molecules in solution. WT CR4/5 (*top* panel), Mut CR4/5 (*middle* panel), and WT CR4/5–TERT complex (*bottom* panel). Red and black lines depict Gaussian functions manually fit to the data with associated *R*^2^. (*C*) Cryo-EM density of human telomerase (EMD-7518) (Nguyen et al. 2018) with the medaka CR4/5 crystal structure (blue, derived from the CR4/5-TRBD structure PDB 4O26) (Huang et al. 2014) manually docked. Approximate locations of each FRET dye are indicated and the distance between these positions within the structural model is indicated together with the estimated FRET value calculated from a Cy3-Cy5 Förster radius of 57 angstroms.

Results from our ensemble chemical probing experiments suggest that the human CR4/5 domain exhibits folding heterogeneity, particularly in the junction region that is proximal to the functionally essential P6.1 stem–loop. To directly detect hTR CR4/5 folding heterogeneity in the presence and absence of TERT protein, and to understand how heterogeneity of hTR CR4/5 secondary structure affects its tertiary conformation, we used a single-molecule Förster resonance energy transfer (smFRET) technique. Single-molecule FRET measures RNA conformation(s) as the distance-dependent energy transfer between a FRET donor (Cy3) and an acceptor (Cy5) dye incorporated into the RNA. FRET probes were strategically incorporated at positions U32 (Cy5) and U70 (Cy3) to establish a dye pair that reports on the physical proximity of the P6 and P6.1 stem–loops (Supplemental Fig. S1). Using this design principle, we created two different FRET constructs: a WT CR4/5 domain and a mutant CR4/5 designed to encourage P6.1 folding (Mut CR4/5) (Fig. 5A). This mutant CR4/5 contains junction linker regions consisting only of adenines intended to constrain its folding landscape to favor the formation of the P6.1 stem. One-dimensional chemical probing with 1M7 and SHAPE-guided modeling of Mut CR4/5 supported the notion that the P6.1 stem within the Mut CR4/5 construct forms more readily compared to WT CR4/5 (Supplemental Fig. S8). Importantly, both the WT and Mut CR4/5 constructs reconstitute active telomerase complexes in vitro (Supplemental Fig. S9). We note that while the Mut CR4/5 construct appeared to show a slight decrease in telomerase reconstitution efficiency, the assembled RNP complexes displayed quantitatively indistinguishable repeat addition processivity values as measured by direct primer extension assays (Supplemental Fig. S9). Single-molecule measurements were made using a solution confocal fluorescence microscope, in which FRET values are extracted from individual freely diffusing molecules as they traverse through the excitation beam. We then collected several thousand FRET values from free and TERT-bound CR4/5 molecules, compiled them into histograms, and fit the data with Gaussian functions to approximate distinct FRET populations.

The WT CR4/5 domain exhibits a substantially heterogeneous FRET profile consisting of at least three unique FRET populations, with the majority of molecules falling into populations centered at higher FRET (∼0.75 and ∼0.9) along with a minor population at lower FRET (∼0.3) (Fig. 5B, top panel). This observation is consistent with our chemical probing data, which suggests the P6.1 stem is not a stably folded motif and that this region of CR4/5 displays structural heterogeneity. Molecules reporting high FRET values likely exist in a conformation in which the P6.1 nucleotides are in close proximity to P6b, while lower FRET states indicated conformations of CR4/5, in which the P6.1 nucleotides are distal from P6b in tertiary space. We then investigated how stabilizing the secondary structure of the P6.1 stem would affect the structural heterogeneity of CR4/5. Whereas the FRET distribution of WT CR4/5 is predominantly represented by two populations reporting higher FRET (0.75 and 0.9) range and marginally low FRET (∼0.3) population, the FRET distribution of Mut CR4/5 appears significantly less heterogeneous, comprised mostly of molecules falling into a single low FRET population (Fig. 5B, middle panel). These observations suggest that stabilizing the P6.1 stem constrains overall structural heterogeneity of CR4/5, shifting the folding landscape toward a low FRET conformation.

Next, we measured the FRET properties of the WT CR4/5 domain after reconstitution with TERT and the hTR template/pseudoknot (t/PK) domain into catalytically active telomerase RNP complexes. Assembly of WT CR4/5 into telomerase RNPs essentially abolishes the apparent heterogeneity of the CR4/5 domain and yields a single low FRET population (∼0.3) (Fig. 5B, bottom panel). This finding suggests that upon telomerase assembly, and consequently the folding of the P6.1 stem motif, the P6.1 and P6b stems are stabilized at an increased distance from each other. The estimated distance (∼65–70 angstroms) between the FRET dyes in an assembled state is consistent with the respective dye label positions modeled in the human telomerase cryo-EM structure (Fig. 5C; Nguyen et al. 2018). This result lends additional support to a human CR4/5 structural transition upon binding to the TERT protein as was proposed for the medaka CR4/5 domain (Huang et al. 2014; Kim et al. 2014).

## DISCUSSION

Telomerase RNPs derived from diverse organisms must assemble upon highly structured telomerase RNA (TR) scaffolds (Zappulla and Cech 2006; Egan and Collins 2012a). TRs possess a multidomain architecture conserved from unicellular ciliates to humans and serve to nucleate the assembly of telomerase complexes through interactions with the telomerase reverse transcriptase (TERT) and other lineage-specific proteins (Romero and Blackburn 1991; Chen et al. 2000; Chen and Greider 2004; Egan and Collins 2010). Despite their essential role in telomerase assembly, it remains unclear how TRs transition from their initial protein-free conformations to the intricate tertiary structures seen in active telomerase complexes (Jiang et al. 2018; Nguyen et al. 2018) and how the nucleotides in the junction of the human CR4/5 affect the structural architecture. In the present study, we use a novel combination of SHAPE-guided RNA modeling and smFRET to demonstrate that the essential P6.1 stem of hTR CR4/5 is not stably folded in vitro and exists as a structural ensemble that is remodeled by the binding of TERT.

The stem terminal element ([STE] stem–loop IV in *Tetrahymena* TR and CR4/5 in hTR) makes a high affinity interaction with TERT (Bley et al. 2011) and, when mutated, abrogates telomerase biogenesis (Mitchell and Collins 2000; Chen et al. 2002), precipitating human disease. In the *Tetrahymena* telomerase RNP, the TR stem–loop IV binds the assembly factor p65, which stabilizes a bent-helix conformation that places the apical loop at the interface of the TRBD and CTE domains of *Tetrahymena* TERT, potentially stabilizing the architecture of TERT (O'Connor and Collins 2006; Stone et al. 2007; Akiyama et al. 2012; Singh et al. 2012; Jiang et al. 2018). Similarly, folding of the TR pseudoknot requires interactions with *Tetrahymena* TERT to stably form and support catalytic activity of telomerase (Mihalusova et al. 2011). In hTR, the H/ACA box proteins (Dyskerin, NOP10, NHP2, and GAR1) regulate telomerase biogenesis and may play a similar role in facilitating the CR4/5 to adopt a conformation that engages the TRBD–CTE interface (Egan and Collins 2012b; Chen et al. 2018). Structural studies of the smaller *Oryzias latipes* (medaka) CR4/5 revealed protein-induced rearrangements of the TWJ motif, rotating the P6.1 stem nearly 180 degrees around the axis of P5 and P6 to clamp upon the TERT RNA-binding domain (TRBD) (Huang et al. 2014; Kim et al. 2014). Presumably, the hTR CR4/5 adopts a similar RNP assembled conformation given it shares invariant nucleotides comprising the P6.1 region and most of the TWJ motif, a notion consistent with the medium-resolution cryo-EM structure of human telomerase (Nguyen et al. 2018). NMR studies demonstrate that pseudouridinylation of the P6.1 stem may alter the structural stability of the P6.1 (Kim et al. 2010; Zemora et al. 2016; Chen et al. 2018); however, the precise role of posttranscriptional modification of hTR in RNA folding and telomerase biogenesis is not firmly established. Moreover, the human TWJ is expanded by ten nucleotides compared to its medaka counterpart and therefore traverses a more complex folding landscape to arrive at its functional RNP state. The role of this expanded junction in human hTR folding has remained enigmatic.

Chemical mapping has been previously used to qualitatively infer hTR structure in its protein-bound and -unbound states (Antal et al. 2002; Zemora et al. 2016). Here, we use chemical data to guide in silico predictions that suggest hTR CR4/5 adopts noncanonical TWJ folds in the absence of TERT protein. Our analysis produces a secondary structure model of medaka CR4/5 consistent with its atomic resolution model (Fig. 2). In contrast, our analysis of human CR4/5 suggests that the P6.1 stem is not stably formed, as it is predicted with notably less abundance as the free energy penalty for its reactive nucleotides are increased within the previously established accurate range of values (Fig. 3; Deigan et al. 2009). An exhaustive mutate-and-map strategy (Kladwang et al. 2011; Tian et al. 2014) of hTR CR4/5 identified base pairing signatures between specific nucleotides in P5 and P6, but was unable to detect Watson–Crick base pairing between nucleotides proposed to form P6.1 (Fig. 4). Notably, two mutations we analyzed by mutate-and-map, C45G and G63C (C287G and G305C, respectively, in full-length hTR) either drastically decrease or abolish 1M7 reactivity of the P6.1 region. C45G (C287G) is a patient-derived hTR mutation in the CR4/5 P6b stem that disrupts RNP assembly and induces aplastic anemia (Yamaguchi et al. 2003). The G63C (G305C) mutation resides in P6.1 and disrupts the same G–C pair as a mutation associated with dyskeratosis congenita (Vulliamy et al. 2011). RNAstructure predictions of these mutants based solely on primary sequence reveal a non-TWJ conformation, in which nucleotides from the P6.1 region pair with nucleotides from the P6a stem (Supplemental Fig. S6). These non-TWJ conformations are consistent with our mutate-and-map data, shedding light on the etiology of diseases arising from mutations in hTR that affect CR4/5 architecture.

**FIGURE 6.**
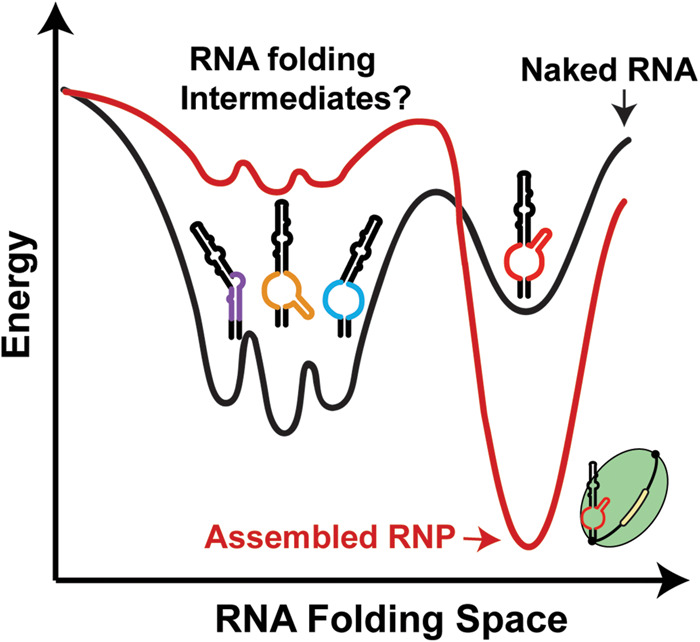
Model describing functional role of CR4/5 folding heterogeneity in human telomerase biogenesis. A schematic depicting a hypothetical folding landscape of the hTR CR4/5 domain. Energy valleys represent unique conformations available to CR4/5. The “depth” of a valley is a conceptual proxy for the stability and relative abundance of a particular RNA conformation. In the folding landscape of the “naked” CR4/5 RNA (black line), there exists a diverse ensemble of TWJ conformations with a small contingent of molecules adopting a fold representative of the canonical P6.1 stem (red TWJ). Upon RNP assembly, the CR4/5 folding landscape becomes dominated by one predominant CR4/5 conformation (red line) because of TERT-induced remodeling of the CR4/5 structure.

The sequence of the P6.1 stem and junction region are strictly conserved across vertebrate TRs. Covariance patterns in CR4/5 suggest evolutionary pressure to maintain the P5 and P6 stems (Chen and Greider 2004), whereas the P6.1 stem lacks any instances of covarying base pairs. Yet, it is known that a stable P6.1 stem is required for TERT binding (Mitchell and Collins 2000; Chen et al. 2002; Bley et al. 2011; Kim et al. 2014). The extreme sequence conservation within P6.1 stem–loop and junction region of hTR suggests the presence of a selective pressure other than preservation of RNA structure alone. Our in vitro smFRET data demonstrate that the majority of the free CR4/5 RNA is in a structure that is not the state found in the TERT-bound state. Using the engineered poly(A) CR4/5 mutant, we observe that the highly conserved nucleotides in the junction are critical in setting up RNA architecture at the junction and that in the absence of competing structures the RNA fold resembles that of the TERT-bound state. This data provides an explanation for the seemingly discordant findings that the junction region is highly conserved but lacks sequence covariation. Namely, that the P6.1 stem is characteristic of the functionally “assembled” state of hTR CR4/5, but in the absence of TERT the junction adopts conformations other than the canonical P6.1 stem (Fig. 6). While the specific identities and functional role(s) of alternate CR4/5 folds remain to be determined, it is conceivable that junction nucleotides may be conserved to preserve RNA structural plasticity required for RNP assembly, as well as to mediate sequence-specific protein interactions that may or may not be present in the fully assembled RNP complex. Another interpretation is that CR4/5 structural heterogeneity limits telomerase assembly, and requires interactions with telomerase proteins to properly assemble the active enzyme. Recently, a study showed that the binding of telomerase-associated protein TCAB1 to hTR positively influences the folding of the P6.1 and P6b stems in an hTR construct lacking the template/pseudoknot domain (Chen et al. 2018). Thus, TCAB1 may enforce proper CR4/5 folding either through direct protein–RNA interactions or potentially mediating access to other binding partners via trafficking hTR to Cajal bodies (Laprade et al. 2020). Future studies using methods such as DMS-MapSeq will permit investigation of how hTR folds in vivo during various stages of telomerase biogenesis, as well as how RNA modifications affect hTR folding (Zubradt et al. 2017).

## MATERIALS AND METHODS

### Preparation of RNAs for chemical probing and in vitro telomerase reconstitution

#### Design and synthesis of RNA chemical probing constructs

Constructs for RNA chemical probing contained the RNA of interest (medaka CR4/5 [nt 170–220] and hTR CR4/5 [nt 243–326]) with additional flanking sequences for normalization purposes in data analysis (described below) and for reverse-transcriptase binding (Kladwang et al. 2014). RNA constructs for chemical probing were iteratively queried on the RNAstructure web server (Reuter and Mathews 2010) and redesigned to discourage base pairing of the flanking sequences with the RNA of interest. Each RNA construct was synthesized by in vitro transcription. The DNA templates were assembled from DNA oligonucleotides designed using the Primerize tool (Tian et al. 2015) and synthesized by IDT (Supplemental Fig. S10; Supplemental Table S2). In the event that a complete DNA template could not be synthesized by one primer assembly reaction using Phusion polymerase (NEB), a “two-piece” scheme was used, in which the products of two separate primer assemblies were used to generate the complete DNA product.

#### In vitro transcription of RNAs

RNA constructs for chemical probing and fragments used for in vitro telomerase reconstitution (hTR CR4/5 [nt 239–328] and hTR t/PK [nt 32–195]) were in vitro transcribed using homemade T7 RNA polymerase (Rio 2013) in RNA polymerase reaction buffer (40 mM Tris-HCl, pH 7.9, 28 mM MgCl_2_, 90 mM DTT, 2 mM spermidine, 1.5 mM each NTP, and 40 U RNasin Plus [Promega]). The reaction was incubated overnight at 37°C followed by the addition of 10 units of TURBO DNase (Thermo Fisher) for 15 min at 37°C. RNA was phenol–chloroform extracted and ethanol precipitated prior to denaturing urea polyacrylamide gel electrophoresis (PAGE) purification. RNAs used in mutate-and-map experiments were transcribed in parallel on 96-well plates and purified using AMPure XP beads (Agencourt). RNA quality was then checked diagnostically by denaturing urea PAGE.

### Structural modeling of RNAs guided by chemical probing data

#### Chemical probing of RNAs

Chemical probing and mutate-and-map experiments were carried out as described previously (Kladwang and Das 2010; Cordero et al. 2014; Kladwang et al. 2014). Briefly, 1.2 pmol of RNA was denaturated at 95°C in 50 mM Na-HEPES, pH 8.0, for 3 min, and folded by cooling to room temperature over 20 min, and adding MgCl_2_ to the desired concentration (1–10 mM). RNA was aliquoted in 15 µL volumes into a 96-well plate and mixed with nuclease-free H_2_O (control), or chemically modified in the presence of 5 mM 1-methyl-7-nitroisatoic anhydride (1M7) (Turner et al. 2013), 25 mM 1-cyclohexyl-(2-morpholinoethyl) carbodiimide metho-*p*-toluene sulfonate (CMCT, Sigma-Aldrich), or 0.25% dimethyl sulfate (DMS, Sigma-Aldrich) for 10 min at room temperature. Mutate-and-map experiments utilized only 1M7 as the chemical modifier and at a 10 mM MgCl_2_. Chemical modification was stopped by adding 9.75 µL quench and purification mix (1.53 M NaCl, 1.5 µL washed oligo-dT beads, Ambion), 6.4 nM FAM-labeled, reverse-transcriptase primer (sequence in Supplemental Table S1), and 2.55 M Na-MES for 1M7 and CMCT reactions, or 50% 2-mercaptoethanol for DMS reactions. RNA in each well was purified by bead immobilization on a magnetic rack and two washes with 100 µL 70% ethanol. RNA was then resuspended in 2.5 µL nuclease-free water prior to reverse transcription.

#### Reverse transcription of modified RNAs and cDNA purification

RNA was reverse-transcribed from annealed fluorescent primer in a reaction containing 1× First Strand Buffer (Thermo Fisher), 5 mM DTT, 0.8 mM dNTP mix, and 20 U of SuperScript III Reverse Transcriptase (Thermo Fisher) at 48°C for 30 min. RNA was hydrolyzed in the presence of 200 mM NaOH at 95°C for 3 min, then placed on ice for 3 min and quenched with 1 volume 5 M NaCl, 1 volume 2 M HCl, and 1 volume 3 M sodium acetate. cDNA was purified on magnetic beads as described previously, then eluted by incubation for 20 min in 11 µL Formamide-ROX350 mix (1000 µL Hi-Di Formamide [Thermo Fisher] and 8 µL ROX350 ladder [Thermo Fisher]). Samples were then transferred to a 96-well plate in “concentrated” (4 µL sample + 11 µL ROX mix) and “dilute” (1 µL sample + 14 µL ROX mix) for saturation correction in downstream analysis. Sample plates were sent to Elim Biopharmaceuticals for analysis by capillary electrophoresis.

#### Analysis of capillary electrophoresis data with HiTRACE

Capillary electrophoresis runs from chemical probing and mutate-and-map experiments were analyzed with the HiTRACE MATLAB package (Yoon et al. 2011). All of the raw data presented in the current study are freely available on the RNA Mapping Database (RMDB IDs: M2CR45_1M7_0000, MCR45_1M7_000, HCR45_1M7_000) (Cordero et al. 2012). Lanes of similar treatment groups (e.g., 1M7 modified) were aligned together, bands fit to Gaussian peaks, background subtracted using the no-modification lane, corrected for signal attenuation, and normalized to the internal hairpin control. The end result of these steps is a numerical array of “reactivity” values for each RNA nucleotide that can be used as weights in structure prediction. For mutate-and-map data sets, each nucleotide is assigned a *Z*-score, calculated as its average reactivity across all mutants divided by the standard deviation (Kladwang et al. 2011). Nucleotides with overall high reactivity across the mutants (average of 0.8 or higher) are ignored in *Z*-score calculation.

#### Data-guided RNA structure prediction

Data-guided secondary structure modeling was performed using the Biers MATLAB package (https://ribokit.github.io/Biers/). Briefly, the Fold function of the RNAstructure suite applied reactivity values as pseudoenergy modifiers to calculate the minimum free energy structure of CR4/5 RNA. Bootstrapping analysis of data-guided structure prediction was performed as described previously (Kladwang et al. 2011; Tian et al. 2014). For mutate-and-map data sets, *Z*-scores were used as pseudoenergy modifiers to calculate a base pairing probability matrix with RNAstructure and to run bootstrapping analysis with Biers. Secondary structures were visualized using the VARNA applet (Darty et al. 2009).

#### Methods for SHAPE modeling

SHAPE-guided predictions were performed with the HiTRACE MATLAB package. The “rna_structure” script was run, while varying the SHAPE slope parameter argument (use the command “open rna_structure” for help) from 1.8 to 5.0 kcal/mol. One hundred bootstrap replicates were performed for each prediction run. Then the results were visualized using the “output_varna” command to produce RNA secondary structure models. For each prediction run, we queried the percent abundance of each canonical human CR4/5 helical element (P5, P6a, P6b, P6.1) and the embedded reference hairpin from among the bootstrapped models. The percent abundance (or bootstrap confidence) of each RNA helix was then recorded under the associated SHAPE slope parameter used to calculate the predictions.

### Telomerase expression and purification

#### In vitro reconstitution of human telomerase

Human telomerase was reconstituted in rabbit reticulocyte lysate (RRL) using the TNT Quick Coupled Transcription/Translation System (Promega) as described previously (Weinrich et al. 1997; Jansson et al. 2019). In LoBind tubes (Eppendorf), 200 µL of TnT quick mix was combined with 5 µg of pNFLAG-hTERT plasmid as well as 1 µM of in vitro transcribed and unlabeled hTR t/PK and CR4/5 fragments. Less abundant dye-labeled CR4/5 was added at 0.1 µM. The reaction was incubated for 3 h at 30°C. 5 µL of 0.5 M EDTA, pH 8.0, were then added to chelate Mg^2+^ ions present in the lysate. Human telomerase was immunopurified via the amino-terminal FLAG tag on hTERT using αFLAG M2-agarose beads (Sigma-Aldrich). Beads contained in 50 µL bead slurry were first washed three times with wash buffer (50 mM Tris-HCl, pH 8.3, 3 mM MgCl_2_, 2 mM DTT, 100 mM NaCl) with 30 sec centrifugation steps at 2350 rcf at 4°C after each wash. The beads were then blocked twice in blocking buffer (50 mM Tris-HCl, pH 8.3, 3 mM MgCl_2_, 2 mM DTT, 500 µg/mL BSA, 50 µg/mL glycogen, 100 µg/mL yeast tRNA) for 15 min under gentle agitation at 4°C followed by 30 sec centrifugation at 2350 rcf and removal of the supernatant. After blocking, the beads were resuspended in 200 µL blocking buffer and added to the telomerase reconstitution reaction in RRL. The beads and lysate were incubated for 2 h at 4°C under gentle agitation. The beads were then pelleted for 30 sec at 2350 rcf and at 4°C and the supernatant was discarded. The beads were then washed three times in wash buffer containing 300 mM NaCl followed by three wash steps in wash buffer containing 100 mM NaCl. A 30 sec centrifugation at 2350 rcf at 4°C was performed between each wash cycle. To elute the enzyme, the beads were incubated in 60 µL elution buffer (50 mM Tris-HCl, pH 8.3, 3 mM MgCl_2_, 2 mM DTT, 750 µg/mL 3× FLAG peptide, 20% glycerol) under gentle agitation at 4°C for 1 h. After elution, the beads were removed by centrifugation at 10,000 rcf through Nanosep MF 0.45 µm filters. 5 µL aliquots were prepared in LoBind tubes (Eppendorf), flash frozen in liquid nitrogen, and stored at −80°C until use.

### Telomerase activity assays

#### ^32^P-end-labeling of DNA primers

A total of 50 pmol of DNA primer was labeled with γ-^32^P ATP using T4 polynucleotide kinase (NEB) in 1× PNK buffer (70 mM Tris-HCl, pH 7.6, 10 mM MgCl_2_, 5 mM DTT) in 50 µL reaction volume. The reaction was incubated for 1 h at 37°C followed by heat inactivation of T4 PNK at 65°C for 20 min. Centrispin columns (Princeton Separations) were used to purify labeled primer.

#### Primer extension assays

Telomerase activity assays of in vitro reconstituted human telomerase were performed using 5 µL purified telomerase in a 15 µl reaction volume brought to 1× activity buffer concentrations (50 mM Tris-HCl, pH 8.3, 50 mM KCl, 1 mM MgCl_2_, 2 mM DTT, 50 nM ^32^P-end-labled primer, and 10 μM of each dATP, dTTP, and dGTP). Reactions were incubated for 90 min at 30°C and quenched with 200 µL 1× TES buffer (10 mM Tris-HCl, pH 7.5, 1 mM EDTA, 0.1% SDS). DNA products were then phenol–chloroform extracted and ethanol precipitated. DNA pellets were resuspended in 1× formamide gel loading buffer (50 mM Tris Base, 50 mM boric acid, 2 mM EDTA, 80% [v/v] formamide, 0.05% [w/v] each bromophenol blue and xylene cyanol), and resolved on a 12% denaturing urea PAGE gel. The gel was then dried and exposed to a storage phosphor screen (GE Healthcare) and scanned using a Typhoon scanner (GE Healthcare). Band intensities were quantified using SAFA and ImageJ (Das et al. 2005; Schneider et al. 2012). The “fraction left behind” (FLB) for a given lane was calculated by summing each repeat addition processivity (RAP) band and all RAP bands below it divided by the total RAP band intensity counts for that lane. The natural logarithm of (1-FLB) was then plotted against repeat number and fitted by linear regression. The slope value of the linear fit was used to determine processivity *R*_1/2_ values from −ln(2)/slope (Latrick and Cech 2010). Total activity was calculated in ImageJ by taking the total intensity of each lane and normalizing to the wild-type lane.

### Preparation of dye-labeled hTR CR4/5 for single-molecule experiments

#### Synthesis of dye-labeled hTR CR4/5 RNA

Synthetic CR4/5 (hTR 239–330) was ordered from Dharmacon as two separate oligonucleotides: Fragment 1 (hTR 239–278) and Fragment 2 (hTR 279–330), each harboring a site-specific aminoallyl modification at the five position of uracil base as indicated in Supplemental Table S1. Oligonucleotides were deprotected in deprotection buffer (100 mM acetic acid, pH 3.6) following the manufacturer's instructions, then ethanol precipitated in the presence of 300 mM sodium acetate, pH 5.2. To enable RNA ligation, Fragment 2 was phosphorylated using T4 PNK (NEB), phenol–chloroform extracted, and ethanol precipitated in the presence of sodium acetate. A total of 10 nmol of each RNA fragment was brought to 100 µL in 0.1 M sodium bicarbonate, pH 9.0, and mixed with an equal volume of a Cy3 or Cy5 Amersham mono-reactive dye pack in DMSO (GE Healthcare). The labeling mix was incubated at 37°C in the dark for 2 h, then ethanol precipitated. Pellets were resuspended in 60 µL buffer A (0.1 M triethylammonium acetate [TEAA], pH 7.5), and HPLC purified on a reversed phase C8 column (Agilent Technologies).

#### Ligation of synthetic RNA fragments

To generate a CR4/5 RNA (hTR 239–328) with fluorescent dyes at positions U274 and U312, a splinted ligation reaction (Akiyama and Stone 2009) containing 800 pmol of Cy3-labeled Fragment 2 (hTR 279-330), 1600 pmol of Cy5-labeled Fragment 1 (hTR 239–278), 1600 pmol of DNA splint (sequence: 5′-AGTGGGTGCCTCCGGAGAAGCCCCGGGCCGAC-3′) in 0.5× T4 DNA ligase buffer (NEB) was brought to 100 µL volume and incubated at 95°C for 5 min and at 30°C for 10 min. A total of 100 µL ligation mix (1.5× T4 DNA ligase buffer, 4000 U T4 DNA ligase [NEB], 2 mM ATP and 1 U/ µL RNAsin Plus [Promega]) was added to the reaction and incubated at 30°C for 18 h. A total of 10 U of TURBO DNase (Thermo Fisher Scientific) was added and the reaction incubated at 37°C for 15 min. The RNA was phenol–chloroform extracted and ethanol precipitated prior to PAGE purification.

### Single-molecule experiments

#### Slide preparation for imaging

Glass micro slides (Gold Seal) were washed by hand with Alconox detergent and warm water, then dried with nitrogen. Sample channels were constructed with Parafilm strips and a plasma-cleaned glass coverslip (Fisher Scientific). Channels were blocked with 10 mg/mL BSA (NEB) for 1 h and washed with imaging buffer (50 mM Tris-HCl, pH 8.3, 50 mM KCl, 1 mM MgCl_2_, 1 mg/ml BSA, 8% glucose, and [±]-6-Hydroxy-2,5,7,8-tetramethylchromane-2-carboxylic acid [Trolox] at saturation). Trolox-containing imaging buffer was generally filtered (0.2 µm) before and after adjusting the pH to 8.3 with NaOH. For imaging, 0.01 volumes of “Gloxy” solution (10 mM Tris-HCl, pH 8.0, 50 mM NaCl, 200 µg/mL catalase, 100 mg/mL glucose oxidase) were added to the imaging buffer.

#### Confocal microscopy of doubly labeled CR4/5 RNA and human telomerase

Data was acquired with a confocal fluorescence microscope with 200-pM-labeled hTR CR4/5 and 50-fold diluted aliquots of in vitro reconstituted labeled human telomerase. A green laser (532 nm) set to 100 µW was used to excite the Cy3 donor dye within the slide channel, and fluorescence from a ∼100 nm^3^ volume was collected through a pinhole and passed on to a dichroic mirror to separate green and red wavelengths. Red and green light were individually detected by avalanche photodiode detectors (APDs) and written to a data file using custom LabView software. Data was collected for 30 min, usually capturing fluorescence from thousands of individual molecules.

#### Analysis of single-molecule data

Using custom MATLAB scripts, the data was thresholded to include only molecules with Cy5 fluorescence one standard deviation above the mean intensity detected by the red (637 nm) APD, as well as corrected for direct Cy5 excitation by green light and dichroic mirror breakthrough. FRET efficiency was calculated in MATLAB with the equation

$$\mathrm{FRET}=I_{A}/(I_{A}+I_{D}),$$

where I_A_ and I_D_ are acceptor and donor intensity, respectively. Histograms were generated using GraphPad Prism. Gaussian approximation of FRET populations was performed by fitting each histogram with a nonlinear regression model, in which the mean of each Gaussian function was constrained to values determined by visual approximation.

## SUPPLEMENTAL MATERIAL

Supplemental material is available for this article.

## Supplementary Material

Supplemental Material
