# Supplementary material for: Folding heterogeneity in the essential human telomerase RNA three-way junction
Source: RNA. 2020 Dec;26(12):1787–800. doi: 10.1261/rna.077255.120 (PMC7668248; doi:10.1261/rna.077255.120)
Supplement: Supplemental Material [file supp_26_12_1787__index.html]

Folding heterogeneity in the essential human telomerase RNA three-way junction — Supplemental Material 

# Folding heterogeneity in the essential human telomerase RNA three-way junction

## Supplemental Material

- Supplemental\_Material.docx
