# Supplementary material for: Folding heterogeneity in the essential human telomerase RNA three-way junction
Source: RNA. 2020 Dec;26(12):1787–800. doi: 10.1261/rna.077255.120 (PMC7668248; doi:10.1261/rna.077255.120)
Supplement: Supplemental Material [file supp_077255.120_Supplemental_Material.docx]

**Supplementary Information**

**Folding heterogeneity in the essential human telomerase RNA three-way junction**

*Running title (50 characters):* Function of telomerase RNA structural heterogeneity

Christina Palka^1*^, Nicholas Forino^2*^, Jendrik Hentschel^1^, Rhiju Das^3,4,5^, Michael D. Stone^1,6^

1. Department of Chemistry and Biochemistry, University of California, Santa Cruz, California 95064, USA.

2. Department of Molecular, Cell, and Developmental Biology, University of California, Santa Cruz, California 95064, USA.

3. Biophysics Program, Stanford University, Stanford, California 94305, USA.

4. Department of Biochemistry, Stanford University, Stanford, California 94305, USA.

5. Department of Physics, Stanford University, Stanford, California 94305, USA.

6. Center for Molecular Biology of RNA, University of California, Santa Cruz, California 95064, USA.

* these authors contributed equally

**Supplementary Figure S1**

**Supplementary Figure S1. Primer extension assay of telomerase reconstituted with WT CR4/5 and dye-labeled CR4/5.** (**A**) Gel illustrating activity and processivity of reconstituted telomerase with WT CR4/5 and dye-labeled CR4/5 (**B**) Quantification of total activity. (**C**) Quantification of repeat addition processivity for telomerase enzymes reconstituted with unlabeled and dye-labeled CR4/5 domain, with indicated R_1/2_ values extracted from the fits to plot of main repeat versus ln(1-FLB), where FLB is Fraction Left Behind (see Materials and Methods for reconstitution and data analysis details). (**D**) CR4/5 dye labeled positions.

**Supplementary Figure S2**

**Supplementary Figure S2. Comparison of structures of the human CR4/5 domain predicted using the RNAstructure software package.** Similar energy values are observed for different structures predicted for the human CR4/5 domain.

**Supplementary Figure S3**

**Supplementary Figure S3. Chemical mapping of medaka and human CR4/5 domains using merged CMCT/DMS data.** Chemical mapping of the medaka (**A**) and human (**B**) CR4/5 domain using merged CMCT/DMS data. Data were collected on TR molecules folded in the presence of 10mM MgCl_2_. Plotted normalized reactivity values are color-coded (red > 0.7, yellow 0.3 - 0.7, and white < 0.3). Bar plot represents experiments conducted in triplicate and error bars are the standard deviation of the three replicates. Color-coded schematic of the reactivity data is shown on the RNAstructure predicted secondary structure.

**Supplementary Figure S4**

**Supplementary Figure S4. Chemical mapping of medaka CR4/5 using 1M7.** Chemical mapping data for the medaka CR4/5 domain collected in the presence of 1mM MgCl_2_ (**A**) mapped onto NMR solution secondary structure and (**B)** tertiary structure (PyMOL) (PDB: 2MHI).

**Supplementary Figure S5**

**Supplementary Figure S5. MgCl_2_ titration of human and medaka CR4/5 using 1M7.**

SHAPE chemical mapping data for Medaka (**A**) and human (**B**) is plotted as a function of the indicated MgCl_2_ concentrations. Bar graphs from three replicates are shown plotted with standard deviation.

**Supplementary Figure S6**

**Supplementary Figure S6. Data-driven structure prediction of select CR4/5 mutants G61C and G63.** The G61C mutation induced an alternate predicted conformation of the CR4/5 domain that resulted in the 5’ buffer annealing to the 3WJ junction region and part of P5 forming a base pairing interaction with the bases normally involved in the P6.1 stem-loop. The G63C mutant caused a dramatic rearrangement in the predicted structure resulting in a totally different structure, with the P6, P5 and 3WJ region all taking on new base-pairing partners. In both constructs the normalization hairpin formed correctly and the mutation made is indicated with a red arrow.

**Supplementary Figure S7**

**Supplementary Figure S7. Z-score plot from mutate-and-map experiments on the hTR CR4/5 domain.** A Z-score plot indicating patterns of mutation-induced changes in chemical reactivity at each nucleotide position across all RNA mutants. The Z-scores are used as weights to guide structure prediction in the RNAstructure software (see Materials and Methods for details).

**Supplementary Figure S8**

**Supplementary Figure S8. SHAPE reactivity of wild-type and mutant hTR CR4/5 domains.** (**A**) 1M7 reactivity data for WT (top panel) and Mut CR4/5 (bottom panel) is shown for data collected in the presence of 10mM MgCl_2_. Bar graphs are data averages from triplicate experiments with standard deviation plotted as error bars. (**B**) SHAPE-guided secondary structure predictions of the hTR WT and Mut CR4/5 domains with reactivity data mapped onto the structural model (Note: only the junction region is shown although the entire hTR CR4/5 construct was included in the modeling).

**Supplementary Figure S9**

**Supplementary Figure S9. Primer extension assay of WT and Mut CR4/5.**

(**A**) Gel illustrating activity and processivity of reconstituted telomerase with WT CR4/5 and Mut CR4/5 (**B**) Quantification of total activity. (**C**) Quantification of repeat addition processivity for telomerase enzymes reconstituted with WT and Mut CR4/5 domain, with indicated R1/2 values extracted from the fits to plot of main repeat versus ln(1-FLB), where FLB is Fraction Left Behind (see Materials and Methods for reconstitution and data analysis details).

**Supplementary Figure S10**

**Supplementary Figure S10. Plate layout for assembling the 84 Mutate-and-Map constructs.** Plate 1, 2, 3, and 4 are all illustrated. For each RNA four primers were used (one from each plate) for the in the primer assembly reaction. By matching the identities of the wells (A1, B1, etc) on each plate, a full-length mutated construct could be assembled (ie - using A1 primers from all plates will make WT CR4/5, using B1 primers from all plates will make mutant C1G, using C1 primers from all will make mutant C2G). Green corresponds to a WT primer for that plate and purple corresponds to a mutant. Only mutant primers (purple) are listed in the M2 oligo table and the primer listed as A1 on each plate lists the WT primer.

**Supplementary Table 1 - Oligonucleotides used in study** (except mutate-and-map)

| **Construct** | **Sequence** |
| --- | --- |
| Human CR4/5 | TTCTAATACGACTCACTATAGGAACGAGAACCCGCCTG |
| Human CR4/5 | GGGTGCCTCCGGAGAAGCCCCGGGCCGACCGCGGCCTCCAGGCGGGTTCTCGTT |
| Human CR4/5 | TCCGGAGGCACCCACTGCCACCGCGAAGAGTTGGGCTCTGTCAGCCGCGGGA |
| Human CR4/5 | GTTGTTGTTGTTGTTTCTTTTGTTGTAAGTACTCCTTACTTTGTTTTCCCGCGGCTGA |
| Medaka CR4/5 | TTCTAATACGACTCACTATAGCAAAAACTGGGAAACGCCGCGGTCA |
| Medaka CR4/5 | AGGGAAACAACAGAGACGAACTCTTCGCAGCAGCCGAGCTGACCGCGGCGTT |
| Medaka CR4/5 | TCGTCTCTGTTGTTTCCCTTTCAAAGCAAGGAGTACTTGCACCAGACACAACACAAT |
| Medaka CR4/5 | GCTGTAAATTGTGTTGTGTCTGGTGCAAGT |
| Mut-1 (lock 7) | TTCTAATACGACTCACTATAGGAACGAGAACCCGCAAAAAAAACGCGGTCGGC |
| Mut-1 (lock 7) | GCGGTGGCAGTGGGTGCCTCCGGAGAAGCCCCGGGCCGACCGCGTT |
| Mut-1 (lock 7) | CCACTGCCACCGCGAAGAGTTGGGCTCTAAAAAAAGCGGGAAAACAAAGTAAGG |
| Mut-1 (lock 7) | GTTGTTGTTGTTGTTTCTTTTGTTGTAAGTACTCCTTACTTTGTTTTCCCGCTTTTTTTA |
| RT FAM primer (medaka) | /56-FAM/AAAAAAAAAAAAAAAAAAAAGCTGTAAATTGTGTTGTGTC |
| RT FAM primer (human) | /56-FAM/AAAAAAAAAAAAAAAAAAAAGTTGTTGTTGTTGTTTCTTT |
| Telo primer for primer extension | TTAGGGTTAGGGTTAGGG |
| Dot Blot Hybridization Oligo | CGG TGG AAG GCG GCA GGC CGA GGC |
| hTR CR4/5 Fragment 1 | GAACCCCGCCUGGAGGCCGCGGUCGGCCCGGGGCU(5-LC-N-U)CUCC3 |
| hTR CR4/5 Fragment 2 | GGAGGCACCCACUGCCACCGCGAAGAGUUGGGC(5-LC-N-U)CUGUCAGCCGCGGGUCUC3 |
| DNA splint for CR4/5 construction | AGTGGGTGCCTCCGGAGAAGCCCCGGGCCGAC3 |

**Supplementary Table 2 - DNA primers used to make Mutate-and-Map templates**

| **PLATE 1** | | |
| --- | --- | --- |
| **WellPosition** | **Name** | **Sequence** |
| *A01* | Lib1-WT | TTCTAATACGACTCACTATAGGAACGAGAACCCGCCTGGAGGCCGCGGTCGGCCCGGGG |
| *B01* | Lib1-C31G | TTCTAATACGACTCACTATAGGAACGAGAAGCCGCCTGGAGGCCGCGGTCGGCCCGGGG |
| *C01* | Lib1-C32G | TTCTAATACGACTCACTATAGGAACGAGAACGCGCCTGGAGGCCGCGGTCGGCCCGGGG |
| *D01* | Lib1-C33G | TTCTAATACGACTCACTATAGGAACGAGAACCGGCCTGGAGGCCGCGGTCGGCCCGGGG |
| *E01* | Lib1-G34C | TTCTAATACGACTCACTATAGGAACGAGAACCCCCCTGGAGGCCGCGGTCGGCCCGGGG |
| *F01* | Lib1-C35G | TTCTAATACGACTCACTATAGGAACGAGAACCCGGCTGGAGGCCGCGGTCGGCCCGGGG |
| *G01* | Lib1-C36G | TTCTAATACGACTCACTATAGGAACGAGAACCCGCGTGGAGGCCGCGGTCGGCCCGGGG |
| *H01* | Lib1-T37A | TTCTAATACGACTCACTATAGGAACGAGAACCCGCCAGGAGGCCGCGGTCGGCCCGGGG |
| *A02* | Lib1-G38C | TTCTAATACGACTCACTATAGGAACGAGAACCCGCCTCGAGGCCGCGGTCGGCCCGGGG |
| *B02* | Lib1-G39C | TTCTAATACGACTCACTATAGGAACGAGAACCCGCCTGCAGGCCGCGGTCGGCCCGGGG |
| *C02* | Lib1-A40T | TTCTAATACGACTCACTATAGGAACGAGAACCCGCCTGGTGGCCGCGGTCGGCCCGGGG |
| *D02* | Lib1-G41C | TTCTAATACGACTCACTATAGGAACGAGAACCCGCCTGGACGCCGCGGTCGGCCCGGGG |
| *E02* | Lib1-G42C | TTCTAATACGACTCACTATAGGAACGAGAACCCGCCTGGAGCCCGCGGTCGGCCCGGGG |
| *F02* | Lib1-C43G | TTCTAATACGACTCACTATAGGAACGAGAACCCGCCTGGAGGGCGCGGTCGGCCCGGGG |
| *G02* | Lib1-C44G | TTCTAATACGACTCACTATAGGAACGAGAACCCGCCTGGAGGCGGCGGTCGGCCCGGGG |
| *H02* | Lib1-G45C | TTCTAATACGACTCACTATAGGAACGAGAACCCGCCTGGAGGCCCCGGTCGGCCCGGGG |
| *A03* | Lib1-C46G | TTCTAATACGACTCACTATAGGAACGAGAACCCGCCTGGAGGCCGGGGTCGGCCCGGGG |
| *B03* | Lib1-G47C | TTCTAATACGACTCACTATAGGAACGAGAACCCGCCTGGAGGCCGCCGTCGGCCCGGGG |
| *C03* | Lib1-G48C | TTCTAATACGACTCACTATAGGAACGAGAACCCGCCTGGAGGCCGCGCTCGGCCCGGGG |
| *D03* | Lib1-T49A | TTCTAATACGACTCACTATAGGAACGAGAACCCGCCTGGAGGCCGCGGACGGCCCGGGG |
| *E03* | Lib1-C50G | TTCTAATACGACTCACTATAGGAACGAGAACCCGCCTGGAGGCCGCGGTGGGCCCGGGG |
| *F03* | Lib1-G51C | TTCTAATACGACTCACTATAGGAACGAGAACCCGCCTGGAGGCCGCGGTCCGCCCGGGG |
| *G03* | Lib1-G52C | TTCTAATACGACTCACTATAGGAACGAGAACCCGCCTGGAGGCCGCGGTCGCCCCGGGG |
| *H03* | Lib1-C53G | TTCTAATACGACTCACTATAGGAACGAGAACCCGCCTGGAGGCCGCGGTCGGGCCGGGG |
| *A04* | Lib1-C54G | TTCTAATACGACTCACTATAGGAACGAGAACCCGCCTGGAGGCCGCGGTCGGCGCGGGG |
| *B04* | Lib1-C55G | TTCTAATACGACTCACTATAGGAACGAGAACCCGCCTGGAGGCCGCGGTCGGCCGGGGG |
| *C04* | Lib1-G56C | TTCTAATACGACTCACTATAGGAACGAGAACCCGCCTGGAGGCCGCGGTCGGCCCCGGG |
| *D04* | Lib1-G57C | TTCTAATACGACTCACTATAGGAACGAGAACCCGCCTGGAGGCCGCGGTCGGCCCGCGG |
| *E04* | Lib1-G58C | TTCTAATACGACTCACTATAGGAACGAGAACCCGCCTGGAGGCCGCGGTCGGCCCGGCG |
| *F04* | Lib1-G59C | TTCTAATACGACTCACTATAGGAACGAGAACCCGCCTGGAGGCCGCGGTCGGCCCGGGC |
| **PLATE 2** | | |
| **WellPosition** | **Name** | **Sequence** |
| *A01* | Lib1-WT | GGGTGCCTCCGGAGAAGCCCCGGGCCGA |
| *D03* | Lib1-T49A | GGGTGCCTCCGGAGAAGCCCCGGGCCGT |
| *E03* | Lib1-C50G | GGGTGCCTCCGGAGAAGCCCCGGGCCCA |
| *F03* | Lib1-G51C | GGGTGCCTCCGGAGAAGCCCCGGGCGGA |
| *G03* | Lib1-G52C | GGGTGCCTCCGGAGAAGCCCCGGGGCGA |
| *H03* | Lib1-C53G | GGGTGCCTCCGGAGAAGCCCCGGCCCGA |
| *A04* | Lib1-C54G | GGGTGCCTCCGGAGAAGCCCCGCGCCGA |
| *B04* | Lib1-C55G | GGGTGCCTCCGGAGAAGCCCCCGGCCGA |
| *C04* | Lib1-G56C | GGGTGCCTCCGGAGAAGCCCGGGGCCGA |
| *D04* | Lib1-G57C | GGGTGCCTCCGGAGAAGCCGCGGGCCGA |
| *E04* | Lib1-G58C | GGGTGCCTCCGGAGAAGCGCCGGGCCGA |
| *F04* | Lib1-G59C | GGGTGCCTCCGGAGAAGGCCCGGGCCGA |
| *G04* | Lib1-C60G | GGGTGCCTCCGGAGAACCCCCGGGCCGA |
| *H04* | Lib1-T61A | GGGTGCCTCCGGAGATGCCCCGGGCCGA |
| *A05* | Lib1-T62A | GGGTGCCTCCGGAGTAGCCCCGGGCCGA |
| *B05* | Lib1-C63G | GGGTGCCTCCGGACAAGCCCCGGGCCGA |
| *C05* | Lib1-T64A | GGGTGCCTCCGGTGAAGCCCCGGGCCGA |
| *D05* | Lib1-C65G | GGGTGCCTCCGCAGAAGCCCCGGGCCGA |
| *E05* | Lib1-C66G | GGGTGCCTCCCGAGAAGCCCCGGGCCGA |
| *F05* | Lib1-G67C | GGGTGCCTCGGGAGAAGCCCCGGGCCGA |
| *G05* | Lib1-G68C | GGGTGCCTGCGGAGAAGCCCCGGGCCGA |
| *H05* | Lib1-A69T | GGGTGCCACCGGAGAAGCCCCGGGCCGA |
| *A06* | Lib1-G70C | GGGTGCGTCCGGAGAAGCCCCGGGCCGA |
| *B06* | Lib1-G71C | GGGTGGCTCCGGAGAAGCCCCGGGCCGA |
| *C06* | Lib1-C72G | GGGTCCCTCCGGAGAAGCCCCGGGCCGA |
| *D06* | Lib1-A73T | GGGAGCCTCCGGAGAAGCCCCGGGCCGA |
| *E06* | Lib1-C74G | GGCTGCCTCCGGAGAAGCCCCGGGCCGA |
| *F06* | Lib1-C75G | GCGTGCCTCCGGAGAAGCCCCGGGCCGA |
| *G06* | Lib1-C76G | CGGTGCCTCCGGAGAAGCCCCGGGCCGA |
| **PLATE 3** | | |
| **WellPosition** | **Name** | **Sequence** |
| *A01* | Lib1-WT | TCCGGAGGCACCCACTGCCACCGCGAAGAGTTGGGCTCTGTCAGCCGCGGGA |
| *C05* | Lib1-T64A | ACCGGAGGCACCCACTGCCACCGCGAAGAGTTGGGCTCTGTCAGCCGCGGGA |
| *D05* | Lib1-C65G | TGCGGAGGCACCCACTGCCACCGCGAAGAGTTGGGCTCTGTCAGCCGCGGGA |
| *E05* | Lib1-C66G | TCGGGAGGCACCCACTGCCACCGCGAAGAGTTGGGCTCTGTCAGCCGCGGGA |
| *F05* | Lib1-G67C | TCCCGAGGCACCCACTGCCACCGCGAAGAGTTGGGCTCTGTCAGCCGCGGGA |
| *G05* | Lib1-G68C | TCCGCAGGCACCCACTGCCACCGCGAAGAGTTGGGCTCTGTCAGCCGCGGGA |
| *H05* | Lib1-A69T | TCCGGTGGCACCCACTGCCACCGCGAAGAGTTGGGCTCTGTCAGCCGCGGGA |
| *A06* | Lib1-G70C | TCCGGACGCACCCACTGCCACCGCGAAGAGTTGGGCTCTGTCAGCCGCGGGA |
| *B06* | Lib1-G71C | TCCGGAGCCACCCACTGCCACCGCGAAGAGTTGGGCTCTGTCAGCCGCGGGA |
| *C06* | Lib1-C72G | TCCGGAGGGACCCACTGCCACCGCGAAGAGTTGGGCTCTGTCAGCCGCGGGA |
| *D06* | Lib1-A73T | TCCGGAGGCTCCCACTGCCACCGCGAAGAGTTGGGCTCTGTCAGCCGCGGGA |
| *E06* | Lib1-C74G | TCCGGAGGCAGCCACTGCCACCGCGAAGAGTTGGGCTCTGTCAGCCGCGGGA |
| *F06* | Lib1-C75G | TCCGGAGGCACGCACTGCCACCGCGAAGAGTTGGGCTCTGTCAGCCGCGGGA |
| *G06* | Lib1-C76G | TCCGGAGGCACCGACTGCCACCGCGAAGAGTTGGGCTCTGTCAGCCGCGGGA |
| *H06* | Lib1-A77T | TCCGGAGGCACCCTCTGCCACCGCGAAGAGTTGGGCTCTGTCAGCCGCGGGA |
| *A07* | Lib1-C78G | TCCGGAGGCACCCAGTGCCACCGCGAAGAGTTGGGCTCTGTCAGCCGCGGGA |
| *B07* | Lib1-T79A | TCCGGAGGCACCCACAGCCACCGCGAAGAGTTGGGCTCTGTCAGCCGCGGGA |
| *C07* | Lib1-G80C | TCCGGAGGCACCCACTCCCACCGCGAAGAGTTGGGCTCTGTCAGCCGCGGGA |
| *D07* | Lib1-C81G | TCCGGAGGCACCCACTGGCACCGCGAAGAGTTGGGCTCTGTCAGCCGCGGGA |
| *E07* | Lib1-C82G | TCCGGAGGCACCCACTGCGACCGCGAAGAGTTGGGCTCTGTCAGCCGCGGGA |
| *F07* | Lib1-A83T | TCCGGAGGCACCCACTGCCTCCGCGAAGAGTTGGGCTCTGTCAGCCGCGGGA |
| *G07* | Lib1-C84G | TCCGGAGGCACCCACTGCCAGCGCGAAGAGTTGGGCTCTGTCAGCCGCGGGA |
| *H07* | Lib1-C85G | TCCGGAGGCACCCACTGCCACGGCGAAGAGTTGGGCTCTGTCAGCCGCGGGA |
| *A08* | Lib1-G86C | TCCGGAGGCACCCACTGCCACCCCGAAGAGTTGGGCTCTGTCAGCCGCGGGA |
| *B08* | Lib1-C87G | TCCGGAGGCACCCACTGCCACCGGGAAGAGTTGGGCTCTGTCAGCCGCGGGA |
| *C08* | Lib1-G88C | TCCGGAGGCACCCACTGCCACCGCCAAGAGTTGGGCTCTGTCAGCCGCGGGA |
| *D08* | Lib1-A89T | TCCGGAGGCACCCACTGCCACCGCGTAGAGTTGGGCTCTGTCAGCCGCGGGA |
| *E08* | Lib1-A90T | TCCGGAGGCACCCACTGCCACCGCGATGAGTTGGGCTCTGTCAGCCGCGGGA |
| *F08* | Lib1-G91C | TCCGGAGGCACCCACTGCCACCGCGAACAGTTGGGCTCTGTCAGCCGCGGGA |
| *G08* | Lib1-A92T | TCCGGAGGCACCCACTGCCACCGCGAAGTGTTGGGCTCTGTCAGCCGCGGGA |
| *H08* | Lib1-G93C | TCCGGAGGCACCCACTGCCACCGCGAAGACTTGGGCTCTGTCAGCCGCGGGA |
| *A09* | Lib1-T94A | TCCGGAGGCACCCACTGCCACCGCGAAGAGATGGGCTCTGTCAGCCGCGGGA |
| *B09* | Lib1-T95A | TCCGGAGGCACCCACTGCCACCGCGAAGAGTAGGGCTCTGTCAGCCGCGGGA |
| *C09* | Lib1-G96C | TCCGGAGGCACCCACTGCCACCGCGAAGAGTTCGGCTCTGTCAGCCGCGGGA |
| *D09* | Lib1-G97C | TCCGGAGGCACCCACTGCCACCGCGAAGAGTTGCGCTCTGTCAGCCGCGGGA |
| *E09* | Lib1-G98C | TCCGGAGGCACCCACTGCCACCGCGAAGAGTTGGCCTCTGTCAGCCGCGGGA |
| *F09* | Lib1-C99G | TCCGGAGGCACCCACTGCCACCGCGAAGAGTTGGGGTCTGTCAGCCGCGGGA |
| *G09* | Lib1-T100A | TCCGGAGGCACCCACTGCCACCGCGAAGAGTTGGGCACTGTCAGCCGCGGGA |
| *H09* | Lib1-C101G | TCCGGAGGCACCCACTGCCACCGCGAAGAGTTGGGCTGTGTCAGCCGCGGGA |
| *A10* | Lib1-T102A | TCCGGAGGCACCCACTGCCACCGCGAAGAGTTGGGCTCAGTCAGCCGCGGGA |
| *B10* | Lib1-G103C | TCCGGAGGCACCCACTGCCACCGCGAAGAGTTGGGCTCTCTCAGCCGCGGGA |
| *C10* | Lib1-T104A | TCCGGAGGCACCCACTGCCACCGCGAAGAGTTGGGCTCTGACAGCCGCGGGA |
| *D10* | Lib1-C105G | TCCGGAGGCACCCACTGCCACCGCGAAGAGTTGGGCTCTGTGAGCCGCGGGA |
| *E10* | Lib1-A106T | TCCGGAGGCACCCACTGCCACCGCGAAGAGTTGGGCTCTGTCTGCCGCGGGA |
| *F10* | Lib1-G107C | TCCGGAGGCACCCACTGCCACCGCGAAGAGTTGGGCTCTGTCACCCGCGGGA |
| *G10* | Lib1-C108G | TCCGGAGGCACCCACTGCCACCGCGAAGAGTTGGGCTCTGTCAGGCGCGGGA |
| *H10* | Lib1-C109G | TCCGGAGGCACCCACTGCCACCGCGAAGAGTTGGGCTCTGTCAGCGGCGGGA |
| *A11* | Lib1-G110C | TCCGGAGGCACCCACTGCCACCGCGAAGAGTTGGGCTCTGTCAGCCCCGGGA |
| *B11* | Lib1-C111G | TCCGGAGGCACCCACTGCCACCGCGAAGAGTTGGGCTCTGTCAGCCGGGGGA |
| *C11* | Lib1-G112C | TCCGGAGGCACCCACTGCCACCGCGAAGAGTTGGGCTCTGTCAGCCGCCGGA |
| *D11* | Lib1-G113C | TCCGGAGGCACCCACTGCCACCGCGAAGAGTTGGGCTCTGTCAGCCGCGCGA |
| *E11* | Lib1-G114C | TCCGGAGGCACCCACTGCCACCGCGAAGAGTTGGGCTCTGTCAGCCGCGGCA |
| **PLATE 4** | | |
| **WellPosition** | **Name** | **Sequence** |
| *A01* | Lib1-WT | GTTGTTGTTGTTGTTTCTTTTGTTGTAAGTACTCCTTACTTTGTTTTCCCGCGGCTGA |
| *C10* | Lib1-T104A | GTTGTTGTTGTTGTTTCTTTTGTTGTAAGTACTCCTTACTTTGTTTTCCCGCGGCTGT |
| *D10* | Lib1-C105G | GTTGTTGTTGTTGTTTCTTTTGTTGTAAGTACTCCTTACTTTGTTTTCCCGCGGCTCA |
| *E10* | Lib1-A106T | GTTGTTGTTGTTGTTTCTTTTGTTGTAAGTACTCCTTACTTTGTTTTCCCGCGGCAGA |
| *F10* | Lib1-G107C | GTTGTTGTTGTTGTTTCTTTTGTTGTAAGTACTCCTTACTTTGTTTTCCCGCGGGTGA |
| *G10* | Lib1-C108G | GTTGTTGTTGTTGTTTCTTTTGTTGTAAGTACTCCTTACTTTGTTTTCCCGCGCCTGA |
| *H10* | Lib1-C109G | GTTGTTGTTGTTGTTTCTTTTGTTGTAAGTACTCCTTACTTTGTTTTCCCGCCGCTGA |
| *A11* | Lib1-G110C | GTTGTTGTTGTTGTTTCTTTTGTTGTAAGTACTCCTTACTTTGTTTTCCCGGGGCTGA |
| *B11* | Lib1-C111G | GTTGTTGTTGTTGTTTCTTTTGTTGTAAGTACTCCTTACTTTGTTTTCCCCCGGCTGA |
| *C11* | Lib1-G112C | GTTGTTGTTGTTGTTTCTTTTGTTGTAAGTACTCCTTACTTTGTTTTCCGGCGGCTGA |
| *D11* | Lib1-G113C | GTTGTTGTTGTTGTTTCTTTTGTTGTAAGTACTCCTTACTTTGTTTTCGCGCGGCTGA |
| *E11* | Lib1-G114C | GTTGTTGTTGTTGTTTCTTTTGTTGTAAGTACTCCTTACTTTGTTTTGCCGCGGCTGA |
